# Rho kinase inhibitor Y-27632 downregulates IL-1β expression in mice with experimental autoimmune myocarditis

**DOI:** 10.1038/s41598-024-60239-8

**Published:** 2024-04-29

**Authors:** Yanjun Li, Ge Gao, Yiru Han, Bingshuai Xiao, Liyuan Shen, Xiangxin Yang, Yangqing Liu, Yaqin Mu, Nianping Zhang, Chunhong Niu, Yuxing Wang

**Affiliations:** 1 Institute of Immunology, Shanxi Datong University School of Medicine, Datong, Shanxi China; 2 Department of Clinical Laboratory, Qilu Hospital of Shandong University, Jinan, Shandong China

**Keywords:** Experimental autoimmune myocarditis, Y-27632, IL-1β, Notch/TLR pathway, Molecular biology, Cardiology

## Abstract

Autoimmune myocarditis is the limited or diffuse inflammation of the myocardium due to dysfunctional cellular and humoral immunity mechanisms. We constructed mouse models of experimental autoimmune myocarditis (EAM) using peptide MyHC-α614-629. On the day after secondary immunization, the mice were intraperitoneally injected with Rho kinase (ROCK) inhibitor Y-27632. On day 21, the cardiac tissues were harvested and weighed. The hearts of EAM mice were significantly enlarged and whitened. Furthermore, body weight (BW) slowly increased during the treatment period, the heart weight (HW) and the ratio of HW/eventual BW were increased, and inflammatory infiltration and fibrosis were aggravated in the myocardial tissue. Y-27632 treatment improved the aforementioned phenotypic and pathological features of EAM mice. Mechanistic analysis revealed a significant increase in Notch1, Hes1, Jag2, Dil1, Toll-like receptor (Tlr) 2, and interleukin (IL)-1β expression in the myocardial tissue of EAM mice. Notably, IL-1β expression was correlated with that of Notch1 and Tlr2. Following Y-27632 treatment, the expression of key target genes of the Notch signaling pathway (*Notch1*, *Hes1*, *Dil1*, and *Jag2*) and *Tlr2* were obviously decreased. Y-27632 treatment also decreased the number of monocytes in the spleen of EAM mice. Thus, ROCK inhibitor Y-27632 exerted a protective effect in EAM mice by downregulating IL-1β expression. This study aimed to provide a reference point for the future treatment of myocarditis in clinical settings.

## Introduction

Myocarditis is the limited or diffuse inflammation of the myocardium caused by noninfectious agents or infectious agents, such as viruses, bacteria, mycoplasma, chlamydia, rickettsia, and fungi^1,2^. Noninfectious myocarditis is mainly classified as either isolated or autoimmune myocarditis. Patients with myocarditis generally present with palpitations, arrhythmias, chest tightness, chest pain, dyspnea, heart failure, and reduced myocardial systolic and diastolic function. Sudden death has also been reported, and myocarditis is the main cause of sudden cardiac death in young people^3,4^. The autoimmune response is an important pathogenic mechanism of myocardial injury, of which the incidence is increasing^5^. Fortunately, clinical trials have confirmed that immunosuppressive therapy relieves the clinical symptoms of myocardiopathy^6^. The experimental autoimmune myocarditis (EAM) mouse model is currently the most popular experimental model for studying the immune mechanism of myocarditis^4,7–9^.

Y-27632, a potent and specific Rho kinase (ROCK) inhibitor, inhibits both ROCK I and ROCK II^10^. It also plays an important role in cardiovascular disease and stem cell culture^11,12^ and is involved in a variety of biological processes, including cell proliferation, adhesion, apoptosis, cell cycle regulation, and angiogenesis^13,14^. ROCKs are aberrantly expressed in a variety of cardiovascular diseases, including cardiac hypertrophy, atherosclerosis, ischemic heart disease, systemic hypertension, and pulmonary hypertension^15,16^.

The Notch signaling family comprises four protein paralogs (Notch1–4) with ligands Delta-like 1, 3, and 4, as well as Jagged 1 and 2. The Notch signaling pathway is highly evolutionarily conserved^17^. Toll-like receptors (TLRs) are ancient pattern recognition receptors that play a vital role in intrinsic immunity^18^. To date, 12 and 10 TLR family members have been identified in mice and humans, respectively^19^. TLR9 is a member of the TLR family. The TLR9 signaling pathway has been reported to promote cardiac inflammation by activating interferon regulatory factor 5 (IRF5) in coxsackievirus B3 (CVB3)-induced viral myocarditis (VMC). Furthermore, interference with the TLR9–IRF5 signaling pathway attenuated VMC damage to cardiomyocytes^20^. TLR4 and nuclear factor kappa B (NF-κB) hyperactivation have been reported in VMC rats, and inhibition of TLR4 and NF-κB expression reduced apoptosis in VMC rat cardiomyocytes, thereby relieving disease symptoms. Furthermore, the expression of TLR4 and phosphorylated NF-κB p65 was significantly increased in CVB3-induced mouse myocardial tissue, as were the expression levels of interleukin (IL)-1β, IL-6, tumor necrosis factor-alpha, and interferon-gamma^21^.

Patients with myocarditis typically exhibit a variety of symptoms, including nonspecific chest pain, chest tightness, dyspnea, and palpitations^22^. These symptoms are sometimes disregarded because they resemble conditions such as coronary artery disease^23^. Therefore, it is crucial to develop effective and specific methods for myocarditis prevention and treatment. Thus, the goal of this study was to explore the role and underlying mechanism of ROCK inhibitor Y-27632 in the treatment of myocarditis using EAM mouse models.

## Results

### Establishment of mouse models of EAM

**Figure 1:**
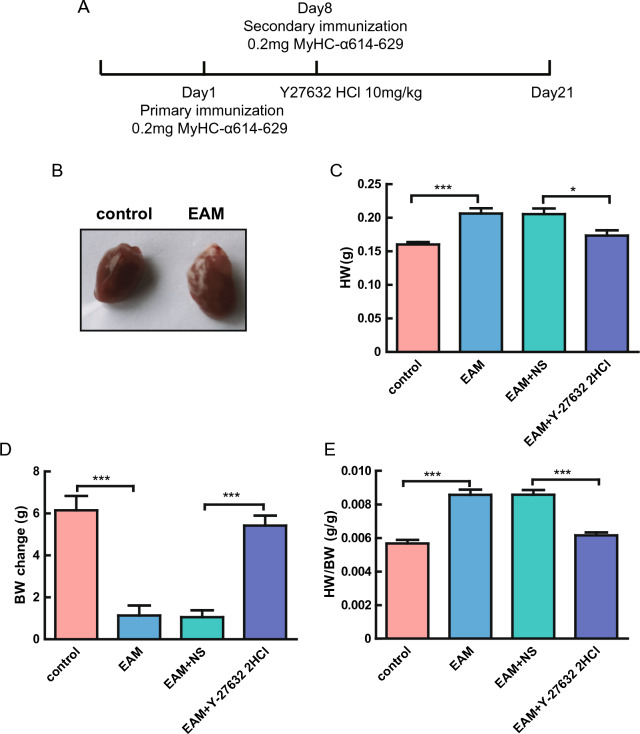
Comparison of BW, HW, and HW/e-BW of mice in the EAM, control, Y-27632 2HCl, and saline groups. (**A**) process of mouse model construction. (**B**) representative photographs of mouse hearts in the EAM and control groups. (**C**) HW of mice in the control, EAM, saline, and Y-27632 2HCl groups. n = 10, *P < 0.05, ***P < 0.001. (**D**) BW of mice in the control, EAM, saline, and Y-27632 2HCl groups. n = 10, ***P < 0.001. (**E**) HW/e-BW of mice in the control, EAM, saline, and Y-27632 2HCl groups. n = 10, ***P < 0.001.

The EAM, saline, and Y-27632 2HCl groups were immunized on days 1 and 8 with an emulsion of 0.2 mg of peptide (MyHC-α614-629) that was injected subcutaneously at multiple points in the groin. The mice were dissected on day 21 (Fig. 1A). The hearts of mice in the EAM group were slightly whitened and significantly enlarged (Fig. 1B). The starting body weight of each mouse was recorded prior to treatment, and the heart weight (HW) and eventual body weight (e-BW) were recorded after dissection on day 21. The HW of the mice in the EAM group was much higher and the BW lower compared with the mice in the control group (Fig. 1C,D, Supplementary Table S1). Additionally, the ratio of HW to e-BW was significantly elevated in the EAM group compared with the control group (Fig. 1E, Supplementary Table S1). The above results tentatively demonstrated the successful establishment of EAM mouse models.

### Y-27632 treatment ameliorated the symptoms of mice with EAM

**Figure 2:**
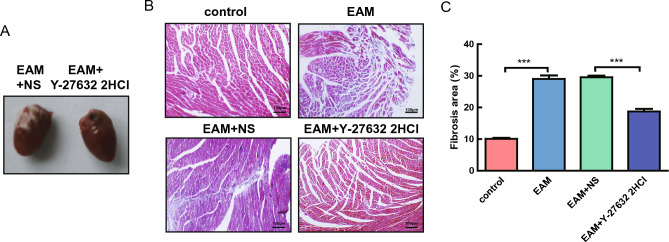
Changes in myocardial tissue fibrosis in mice in the EAM, control, Y-27632 2HCl, and saline groups. (**A**) representative plots of mouse hearts in the Y-27632 2HCl and saline groups. (**B**) representative plots of Masson trichrome staining of mouse myocardial tissues. (**C**) proportion of myocardial tissue fibrosis in mice in the control, EAM, saline, and Y-27632 2HCl groups. n = 8, ***P < 0.001.

Compared with the saline group, the HW of the Y-27632 2HCl group was significantly reduced and the BW significantly increased (Fig. 1C,D, Supplementary Table S2). The ratio of HW/e-BW was much lower in the Y-27632 2HCl group compared with the saline group (Fig. 1E, Supplementary Table S2). The whitened hearts were improved and the heart volume was significantly reduced in the Y-27632 2HCl compared with the saline group (Fig. 2A). Masson trichrome staining was performed on sections of mouse myocardial tissue to histologically evaluate the extent of myocardial tissue fibrosis. The results showed that the percentage of blue area in the EAM group was notably higher compared with the control group, indicating a significant increase in myocardial fibrosis in EAM mice. The Y-27632 2HCl group showed an obvious decrease in the percentage of blue area compared with the saline group, indicating that Y-27632 alleviated myocardial fibrosis in mice (Fig. 2B,C). These experimental results confirmed that Y-27632 2HCl intervention significantly improved the symptoms in EAM mice.

### Y-27632 treatment alleviated inflammation in mice with EAM

**Figure 3:**
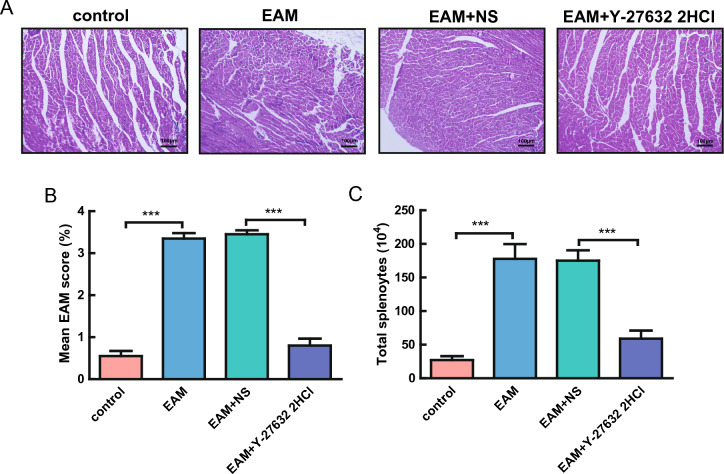
Y-27632 2HCl treatment effectively alleviated the inflammation of myocardial tissue in EAM mice. (**A**) Representative graph of HE staining of myocardial tissue in mice. (**B**) Inflammatory infiltration score of myocardial tissue in mice in the control, EAM, saline, and Y-27632 2HCl groups. n = 8, ***P < 0.001. (**C**) number of mononuclear cells in the spleen of mice in the control, EAM, saline, and Y-27632 2HCl groups. n = 8, ***P < 0.001. *EAM* experimental autoimmune myocarditis.

Hematoxylin and eosin (HE) staining and mononuclear cell culture were performed to determine the extent of myocardial inflammation in the mouse groups. HE staining confirmed that the inflammation score of myocardial tissue was relatively higher in the EAM group compared with the control group, indicating a significant increase in inflammatory infiltration in EAM mice. Additionally, the inflammation score was significantly decreased following Y-27632 2HCl treatment, suggesting that Y-27632 alleviated inflammatory infiltration (Fig. 3A,B).

The spleen is the largest peripheral organ of the immune system, and the proportion or number of mononuclear cells (lymphocytes and monocytes) indirectly and effectively reflects the inflammatory condition of an organism. Therefore, the number of mononuclear cells in the mouse spleen is an effective representation of the condition of the mouse. Our experimental results revealed that the number of mononuclear cells in the spleen of EAM mice was considerably increased compared with the control group and was markedly decreased following Y-27632 2HCl treatment, implying the alleviation of inflammation in the myocardial tissue of mice (Fig. 3C).

### Y-27632 significantly decreased the expression of pro-inflammatory factor IL-1β

**Figure 4:**
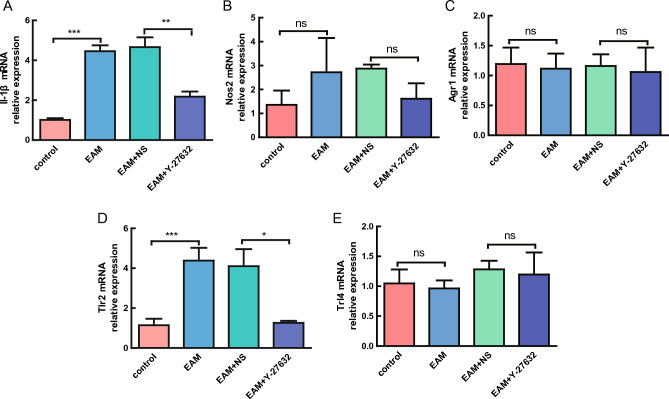
Expression levels of pro-inflammatory factors and key genes of the TLR signaling pathway as determined by qRT-PCR. (**A**–**C**) qRT-PCR results of the expression of IL-1β, Nos2, and Agr1. n = 8, **P < 0.01, ***P < 0.001. (**D**–**E**) qRT-PCR results of the expression of Tlr2 and Tlr4. n = 8, *P < 0.05, ***P < 0.001. *ns* not significant.

We used quantitative reverse transcription (qRT)-polymerase chain reaction (PCR) assays to determine the levels of expression of IL-1β, Nos2, Agr1, and TLRs. The results showed increased expression of IL-1β and Nos2 in the EAM group compared with the control group, but the increase in Nos2 expression was not statistically significant. Following Y-27632 2HCl treatment, IL-1β and Nos2 expression were decreased compared with the saline group, but only the decrease in IL-1β expression was statistically significant (Fig. 4A–C).

The TLR signaling pathway was associated with inflammation, and the expression levels of its related genes (*Tlr2* and *Tlr4*) were determined by qRT-PCR. TRL2 was significantly upregulated in the EAM group compared with the control group and was significantly reduced following Y-27632 2HCl treatment (Fig. 4D,E). We speculated that, logically, symptom improvement in EAM mice following Y-27632 2HCl treatment might be due to the decreased expression of pro-inflammatory factor IL-1β and the inhibition of TLR activity.

### Inhibition of ROCK activity beneficially alleviated IL-1β upregulation caused by Notch signaling pathway activation

**Figure 5:**
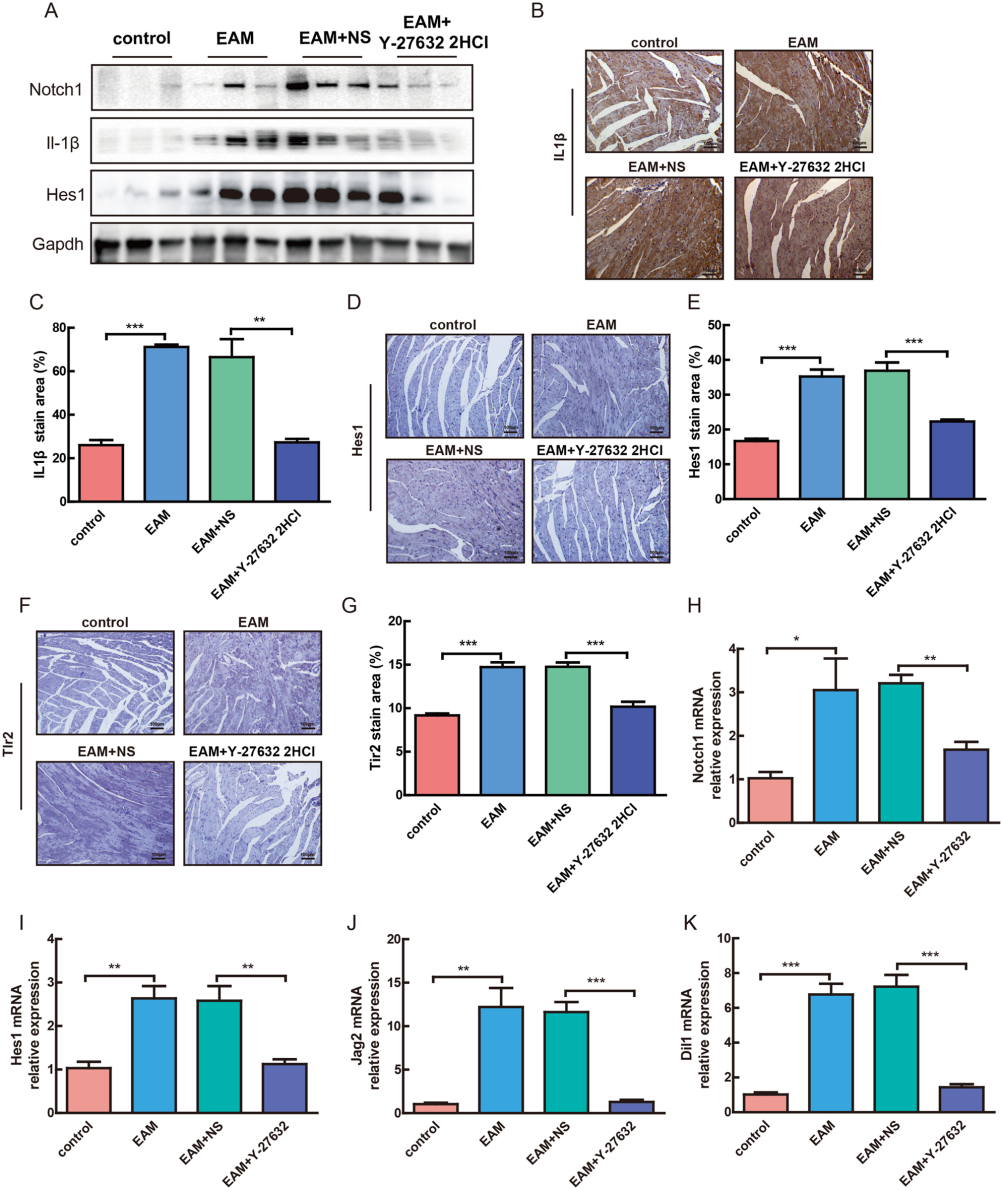
Inhibition of ROCK activity beneficially alleviated the upregulation of IL-1β caused by Notch signaling pathway activation. (**A**) western blot to detect the expression of Notch1, IL-1β, and Hes1. n = 3. (**B,D,F**) representative staining plots of mouse myocardial tissue for IL-1β, Hes1, and Tlr2 in the control, EAM, saline, and Y-27632 2HCl groups. (**C,E,G**) IHC detection of IL-1β-, Hes1-, and Tlr2-positive areas in myocardial tissue in the control, EAM, saline, and Y-27632 2HCl groups. n = 8, **P < 0.01, ***P < 0.001. (**H–K**) qRT-PCR results of the expression of Notch1, Hes1, Jag2, and Dil1 in the control, EAM, saline, and Y-27632 2HCl groups. n = 8, **P < 0.01, ***P < 0.001.

**Figure 6:**
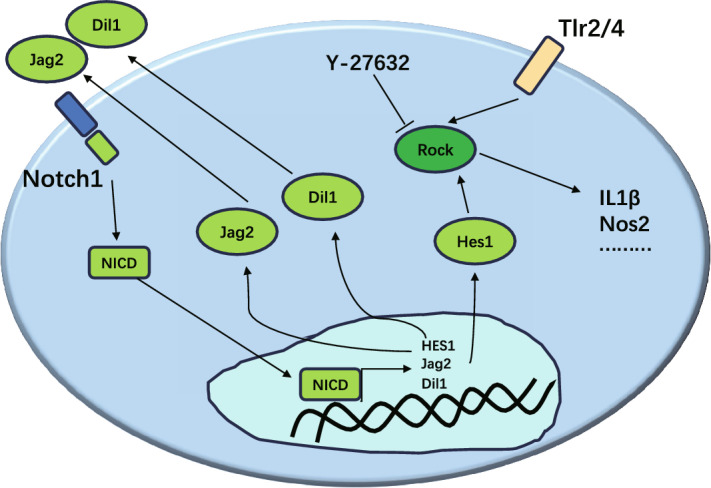
Proposed model of the Notch-ROCK pathway and its biological significance. The cytoplasmic form of Notch1, independent of its transcriptional activity, triggers caspase-mediated cleavage (activation) of ROCK, which promotes increased expression of inflammatory factors, resulting in the exacerbation of myocardial inflammation.

Y-27632 2HCl treatment was found to reduce the expression of inflammatory factor IL-1β in EAM mice. The expression of IL-1β has been reported as associated with Notch signaling pathway activity. Our above results suggested that Notch signaling might also be associated with the inhibition of TLR activity. To confirm this, we performed western blot analysis to determine the expression of Notch1, IL-1β, and Hes1. The results revealed the increased expression of Notch1, IL-1β, and Hes1 in the myocardial tissue of EAM mice, all of which were significantly decreased following treatment with Y-27632 2HCl (Fig. 5A). These findings were mirrored in the immunohistochemistry (IHC) results (Fig. 5B–G). To further confirm these results, the expression levels of the target genes of the Notch signaling pathway (*Notch1*, *Hes1*, *Dil1*, and *Jag2*) were determined by qRT-PCR assays. The results showed that the expression levels of all the target genes were significantly upregulated in the myocardial tissue of EAM mice and downregulated following Y-27632 2HCl treatment (Fig. 5H–K). Collectively, our findings indicated that Y-27632 2HCl treatment of EAM mice reduced the expression of inflammatory factor IL-1β (Fig. 6).

## Discussion

Myocarditis is an inflammatory heart disease that can be either infectious or noninfectious^2^. Noninfectious myocarditis is induced by toxic substances, drugs (e.g., immune checkpoint inhibitors), systemic immune diseases, and other factors. This investigation only focused on autoimmune myocarditis.

Y-27632 is the most common ROCK inhibitor and is frequently utilized in cardiovascular disease studies^24,25^. ROCK plays important roles in various biological processes, such as cell proliferation, metastasis, and apoptosis, as well as cell cycle regulation^26^. Currently, the EAM mouse model is the most commonly used experimental model for determining the immune mechanism of myocarditis pathogenesis. This study investigated the effect of Y-27632 treatment in EAM mice. We found that the EAM group had a lower BW, higher HW, and higher HW/e-BW ratio compared with the control group. Additionally, inflammatory infiltration and fibrosis were aggravated in the myocardial tissue of EAM mice, and the number of mononuclear cells in the spleen was also considerably increased. Following Y-27632 treatment, BW increased, HW and HW/e-BW decreased, the extent of inflammatory infiltration and fibrosis in myocardial tissues were reduced, and the number of mononuclear cells in the spleen was obviously decreased. These findings confirmed the role of Y-27632 in the treatment of EAM mice.

The aforementioned outcomes demonstrated that Y-27632 alleviated the symptoms in EAM mice. The expression of IL-1β, Nos2, Agr1, and TLRs was investigated to elucidate the mechanism of action of Y-27632. IL-1β, Nos2, and Tlr2 expression levels were higher in the myocardial tissue of EAM mice and lower following Y-27632 treatment. Because the difference in Nos2 expression was not statistically significant, we focused on IL-1β and Tlr2 expression. Abnormal IL-1β expression is related to the activity of the Notch/TLR signaling pathway, which is crucial to the immune system^27,28^. Our study results showed the consistent expression of IL-1β, Tlr2, and Notch1 in the myocardial tissues of mice. Additionally, changes in the expression of Notch signaling pathway target genes (*Notch1*, *Hes1*, *Dil1*, and *Jag2*) were observed. Thus, it follows that Y-27632 ameliorates the symptoms of EAM mice by suppressing IL-1β expression.

Many research group has found that the ROCK inhibitor fasudil plays an important role in the treatment of myocarditis^29^. Accordingly, ROCK inhibition beneficially reduces the symptoms of myocarditis. This finding offers a specific reference point for the future clinical management of myocarditis, and we believe that ROCK inhibitors will become more significant in the field of medicine.

The incidence of myocarditis is increasing, and its harmful effects must not be underestimated. The causes of myocarditis are also diverse. Despite great progress in myocarditis research, attention still needs to be paid to its sequelae and prognosis. Moreover, the multiple etiologic causes of this disease make research staged and complex. Many issues need addressing, such as the low value of current imaging methods in patients with chronic inflammatory cardiomyopathy and persistent low-grade inflammation, the low accuracy of viral genomic testing of cardiac samples, and the need for continued improvement in viral testing. There is also room for extensive improvement in research on markers of myocarditis.

This study had some limitations. Although it focused on changes in the histologic and pathological features of the heart as well as changes in key inflammatory factors, it did not pay special attention to the toxic effects of Y-27632 on the animals themselves, changes in cardiac function, screening for important regulatory factors in the context of big data, and follow-up of the prognosis of myocarditis treated with Y-27632. Therefore, future studies should focus on the toxic effects of the inhibitor, cardiac function, and prognosis.

## Materials and methods

### Ethics approval

All animal studies were conducted according to the protocol approved by the Animal Care and Use Committee of Shanxi Datong University Biomedical Research Ethics Review Committee.

### Study subjects

Forty BALB/c male mice aged 5–6 weeks were purchased from Beijing HFK Bio-Technology Co., Ltd. The animals were housed in controlled environmental conditions (temperature, 20–22 °C; humidity, 50–55%) under a reversed 12 h light/dark cycle with access to food and water ad libitum. Animal maintenance, treatments, and experimental procedures were conducted according to Medical and Biological Ethics Committee regulations and approved by the Medical and Biological Ethics Committee of Shanxi Datong University.

### Mouse model construction

The 40 BALB/c mice were randomly divided into control, EAM, saline, and Y-27632 2HCl group. The control group was injected subinguinally with 200 μl of adjuvant dilution (adjuvant: saline = 1:1) on days 1 and 8. The EAM, saline, and Y-27632 2HCl groups were injected subinguinal with 200 μl of an emulsion containing 0.2 mg of peptide (MyHC-α614-629) on days 1 and 8. Mice in the saline group received intraperitoneal injections of 200 μl of saline every other day starting from day 8 until day 21, and mice in the Y-27632 2HCl group received intraperitoneal injections of Y-27632 2HCl (10 mg/[kg day]) every other day starting from day 8 until day 21.

### Mononuclear cell culture

Whole mouse spleen was fully ground using a grinder, washed 5–8 times with phosphate-buffered saline, filtered through a cell strainer, and centrifuged at 1000 rpm/min for 5 min. Then, 10 ml of Dulbecco modified Eagle was added, and the cell suspension was transferred to a 10 cm culture dish and cultured for 5 days at 37 °C with 5% CO_2_. Thereafter, the number of mononuclear cells cultured from each mouse spleen was counted, and graphs were constructed to compare the number of mononuclear cells in each of the four groups.

### HE and IHC staining

HE staining: The specific steps were detailed in the references^30^. Paraffin sections of myocardial tissue underwent HE staining. Then, four random fields were photographed under the microscope for each section. Two pathologists blinded to the treatment group scored the inflammation levels according to the amount of inflammatory infiltrate observed as follows: 0, 0–19%; 1, 20–39%; 2, 40–59%; 3, 60–79%; and 4, 80–100%. Then, graphs were constructed for comparative analysis.

Paraffin sections of myocardial tissue underwent IHC staining with primary antibodies (Hes1, Tlr2, and IL-1β) and secondary antibodies (anti-rabbit and anti-mouse) purchased from Shandong Huaan Biotechnology Co., Ltd. Immunohistochemistry: The details of the steps were described in the references^30^. Then, four random fields were photographed under the microscope for each section, and the proportion of brown (positively stained) areas was analyzed using Image statistical software.

### Masson trichrome staining

Paraffin sections of myocardial tissue underwent dewaxing and hydration. Then, they were stained with Weigert's iron hematoxylin for 7 min, followed by Masson bluing reagent for 5 min, washed in distilled water for 1 min, and placed in phosphomolybdic acid for 2 min and aniline blue solution for 2 min. Next, they were dehydrated and decolorized with alcohol for 3 min, cleared in xylene, and mounted in neutral balsa. Then, four random fields were photographed under the microscope. The proportion of blue areas was analyzed for each section using Image statistical software.

### Western blotting

TRIzol reagent or RIPA buffer was used to extract the total protein. Then, 40 μg of the extracted protein was denatured for western blotting, which was performed as previously described^30^. Imaging was performed using the Bio-Rad ChemiDoc XRS Chemiluminescence imaging system. The primary antibodies used were Notch1 (rabbit, 1:1000), GAPDH (rabbit, 1:3000), Hes1 (rabbit, 1:500), and IL-1β (mouse, 1:1000). The secondary antibody was anti-rabbit or anti-mouse (1:5000), as appropriate. Notch1 and GAPDH were purchased from Cell Signaling Technology, and Hes1, IL-1β, and secondary antibodies (ani-rabbit and anti-mouse) were purchased from Shandong Huaan Biotechnology Co., Ltd.

### RNA extraction and qRT-PCR

RNA was extracted from myocardial tissue using TRIzol reagent, and qRT-PCR was performed as previously described^31,32^. The housekeeping gene Gapdh was selected as the internal control, and the relative expression levels of the target genes were determined using the 2^−∆∆Ct^ method. The primer sequences are listed in Supplementary Table S3.

### Statistical analysis

The data were imported into GraphPad Prism 5.0 as the mean ± standard error of the mean or mean ± standard deviation. Individual experimental mice were removed if the data dispersion is large. Differences between two groups were compared using *t*-tests. *P*-values < 0.05 were considered statistically significant.

### Supplementary Information

Supplementary Information 1.

Supplementary Information 2.

Supplementary Information 3.

## Data Availability

The datasets used during the current study are available from the authors on reasonable request.
