# Supplementary material for: Rho kinase inhibitor Y-27632 downregulates IL-1β expression in mice with experimental autoimmune myocarditis
Source: Sci Rep. 2024 Apr 29;14:9763. doi: 10.1038/s41598-024-60239-8 (PMC11058197; doi:10.1038/s41598-024-60239-8)

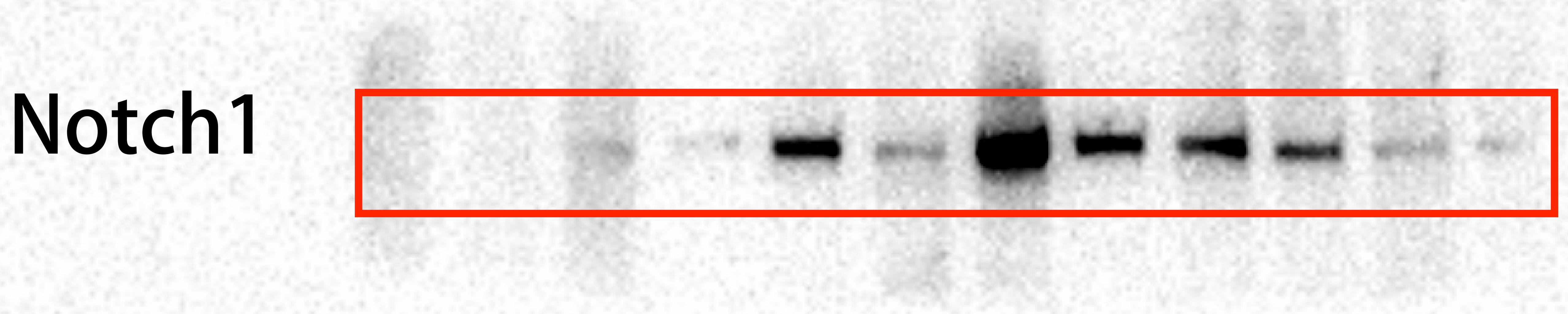
Notch1

Notch1

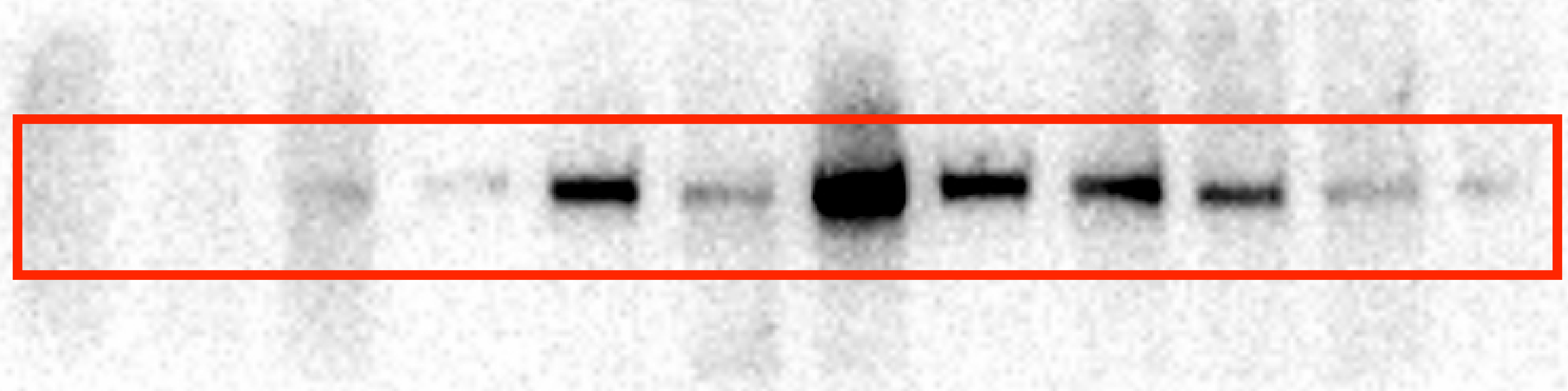

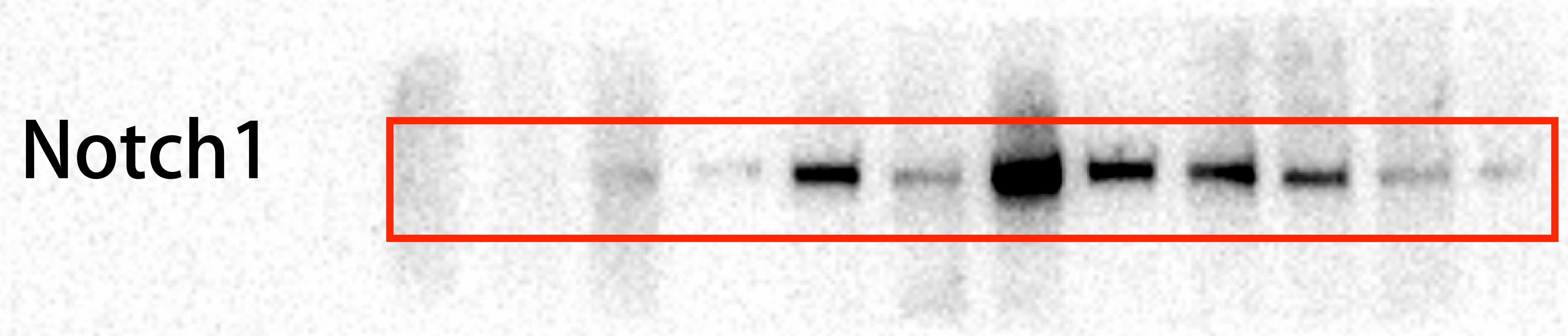
Notch1

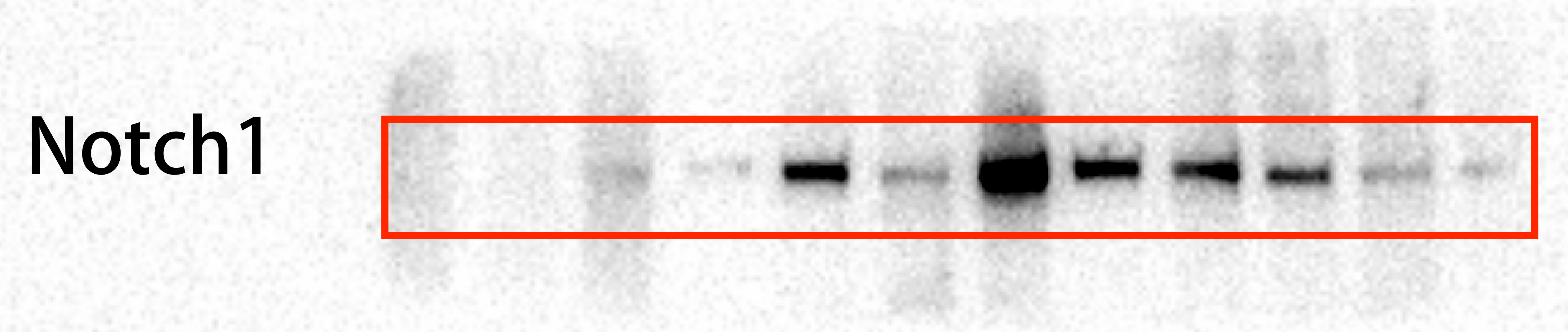
Notch1

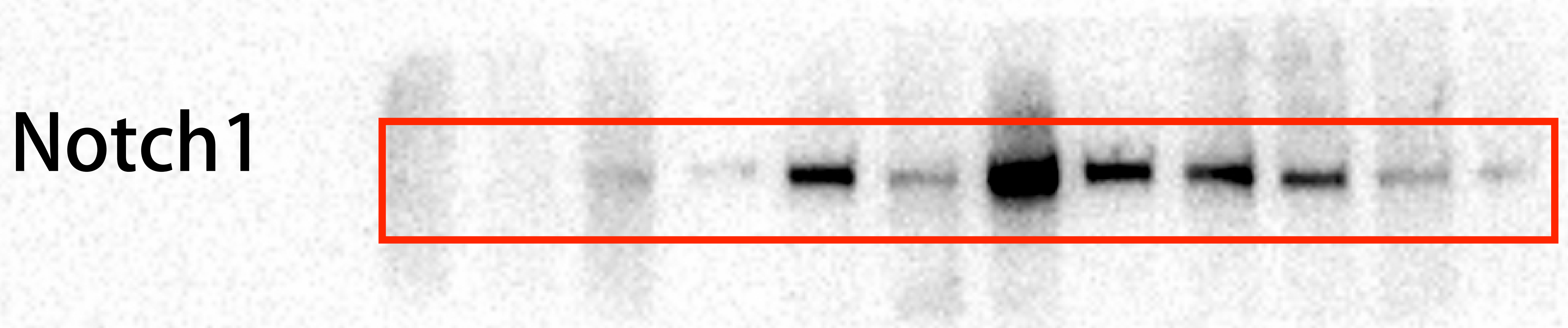
Notch1

Notch1

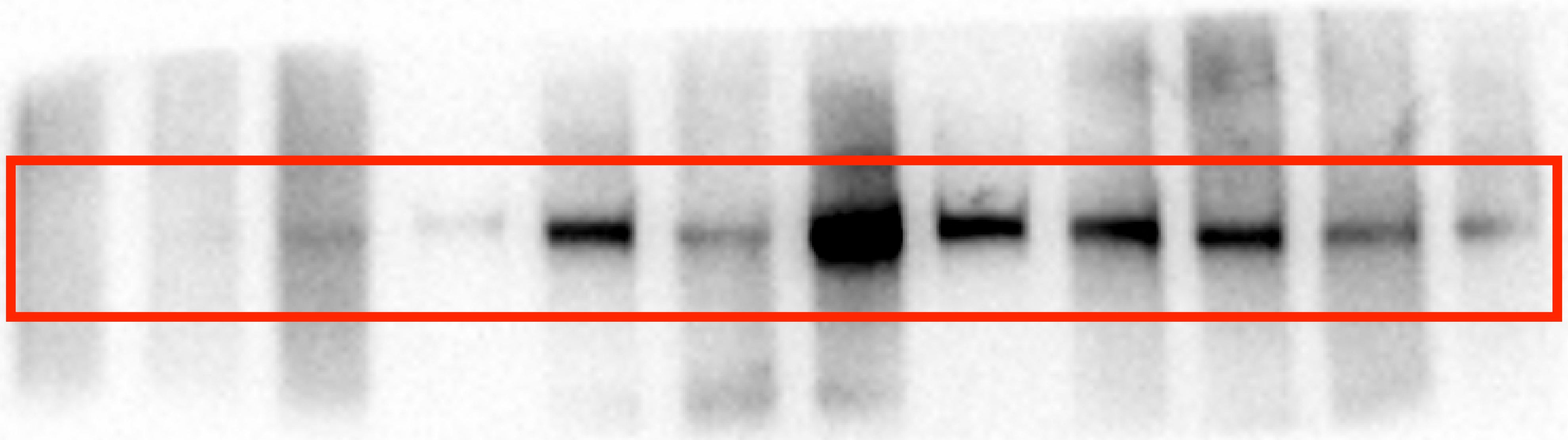

IL-1 β

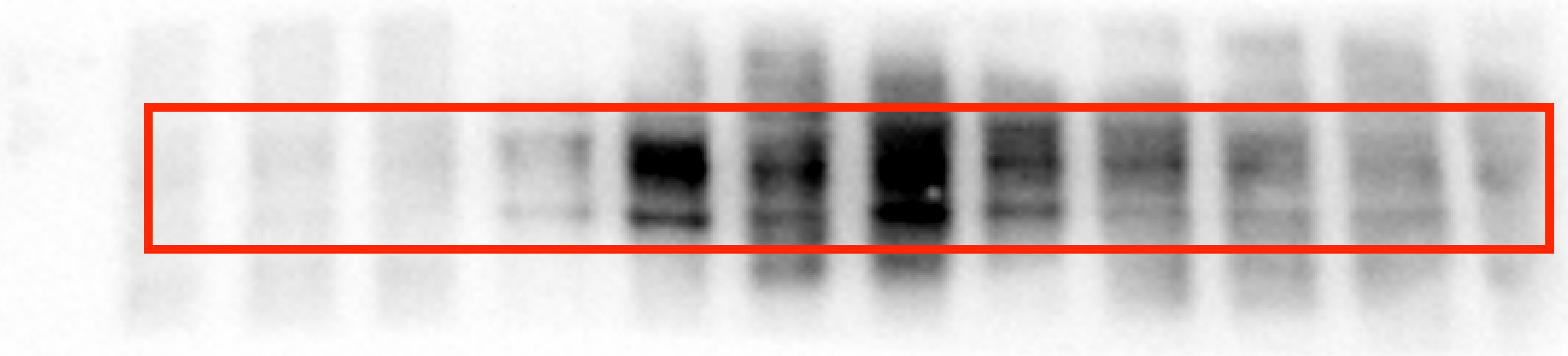

IL-1 β

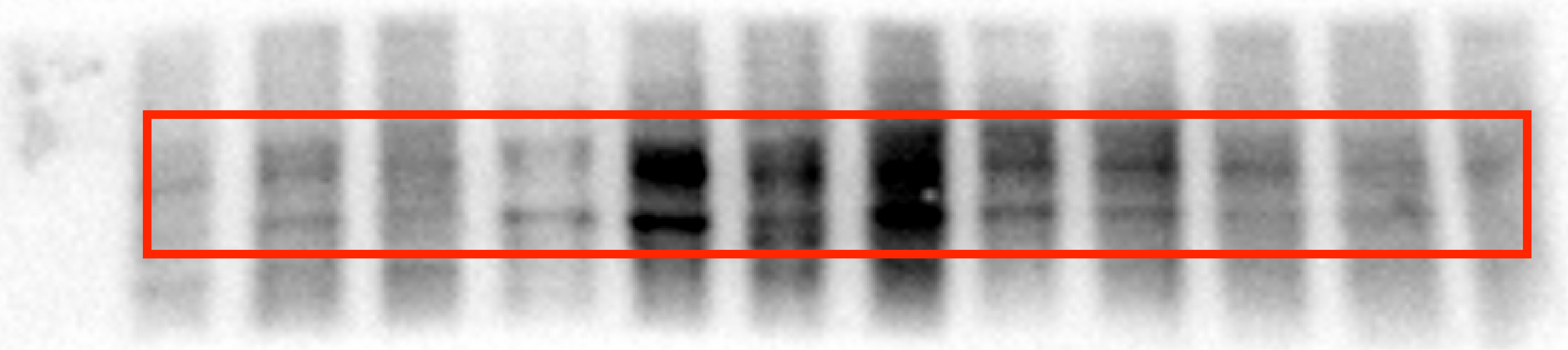

IL-1 β

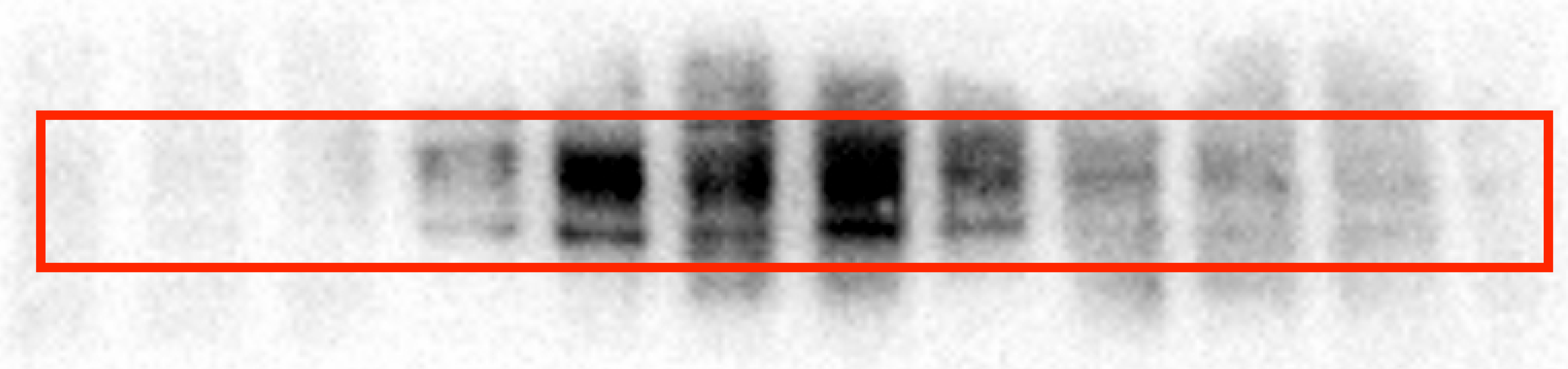

IL-1 β

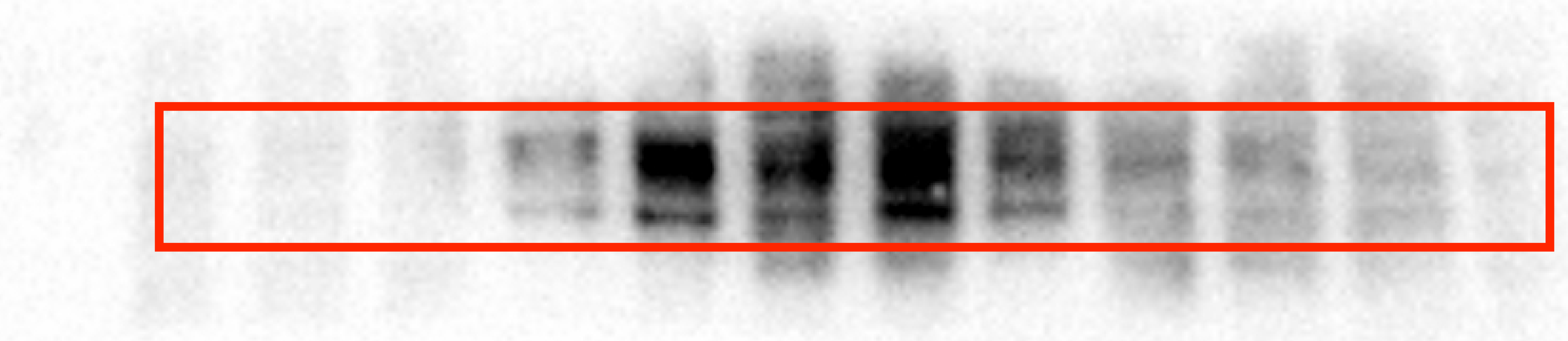

IL-1 β

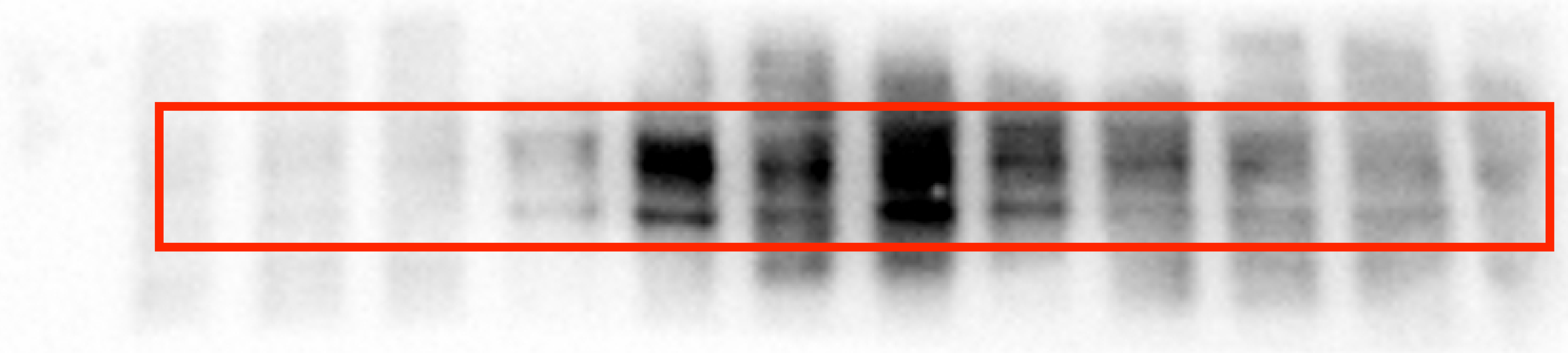

Hes1

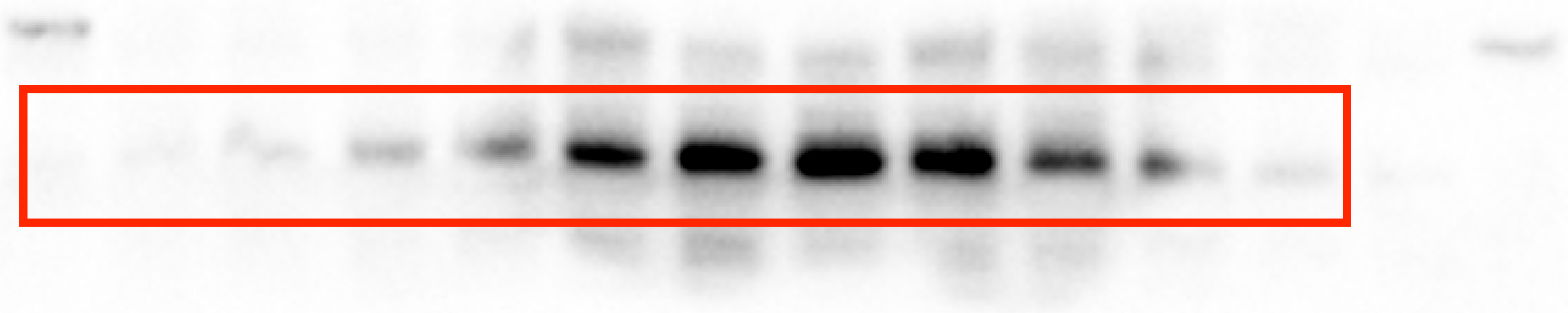

Hes1

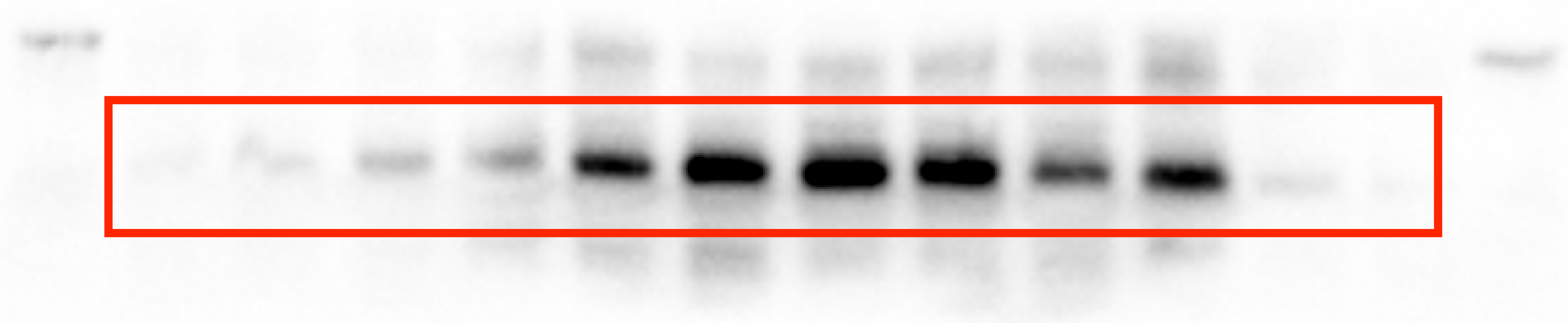

Hes1

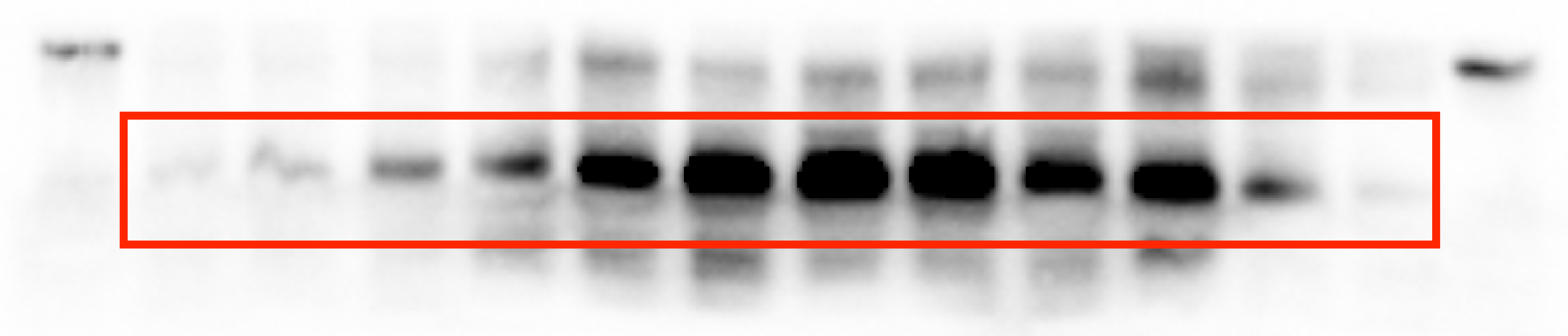

Hes1

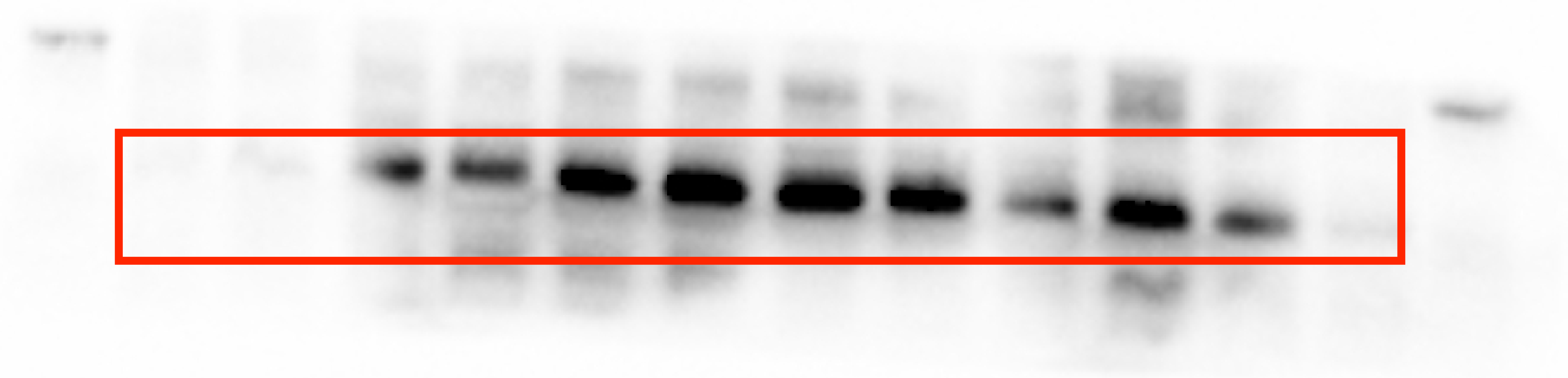

Hes1

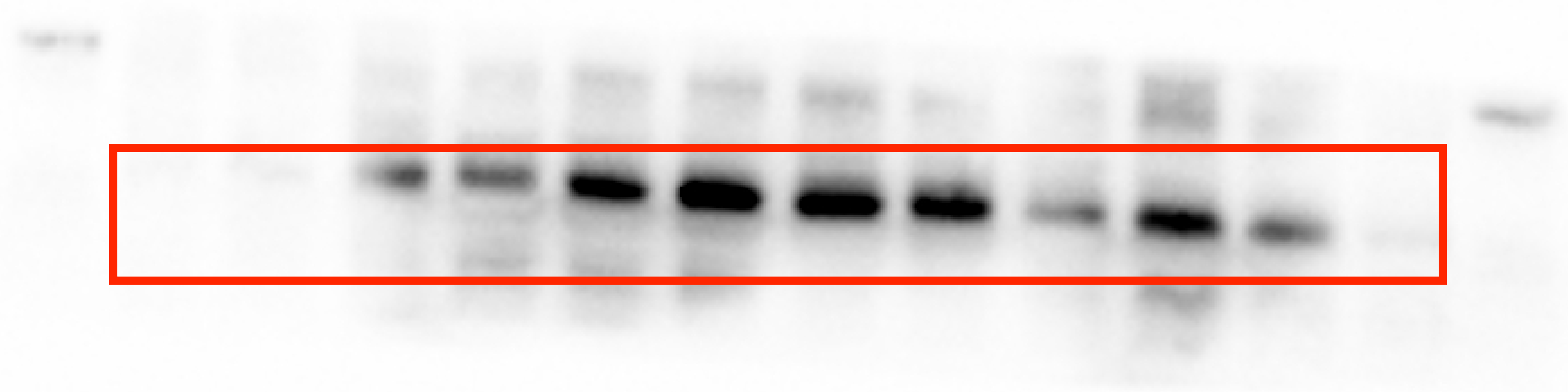

Gapdh

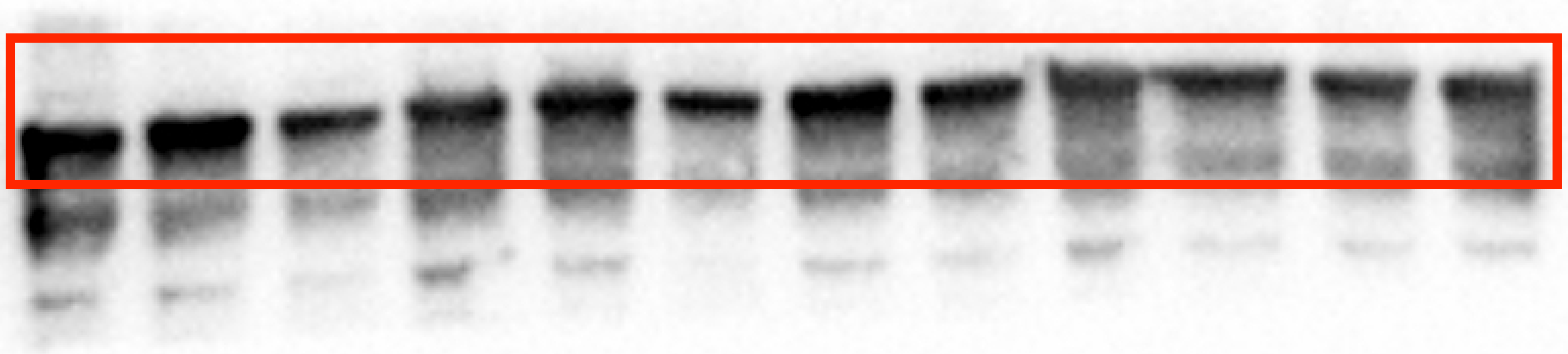

Gapdh

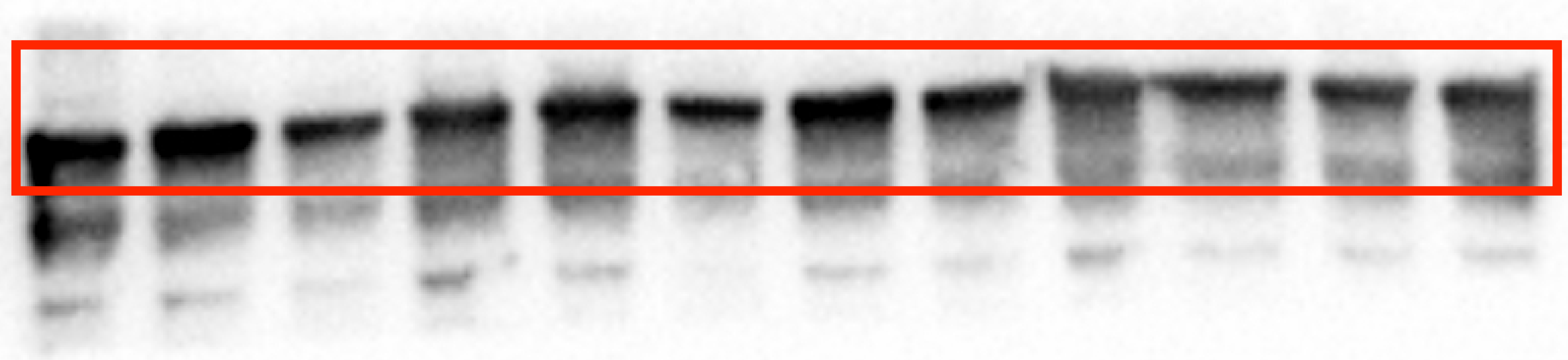

Gapdh

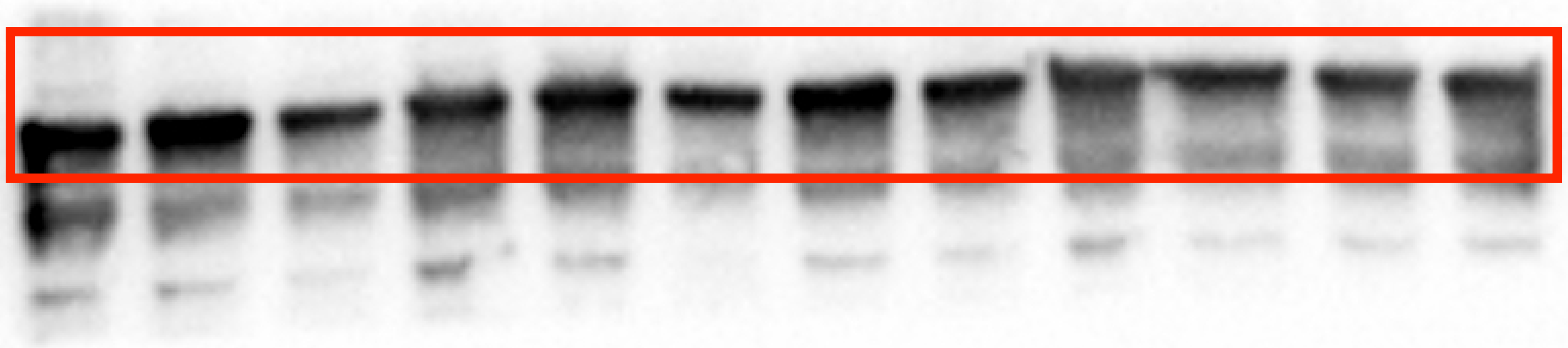

Gapdh

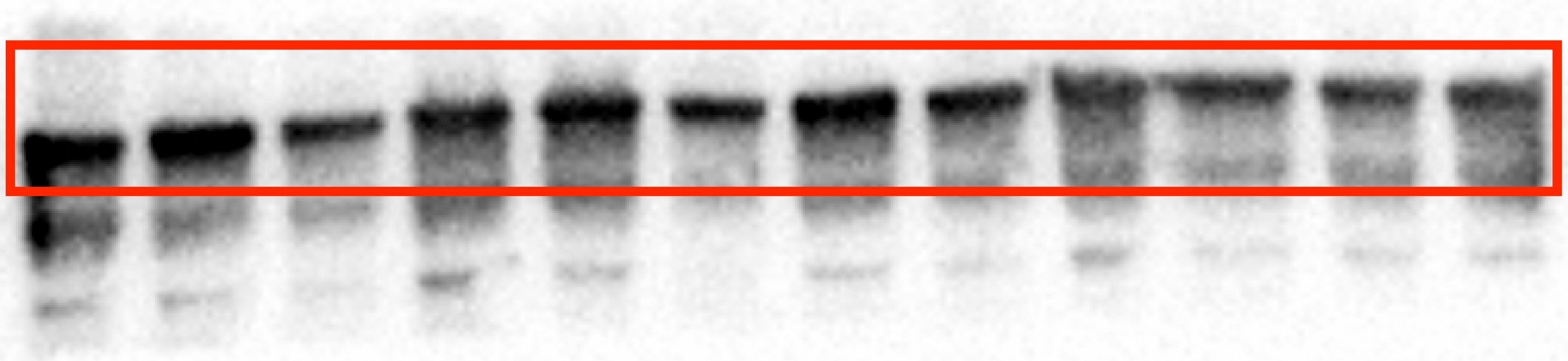

Gapdh

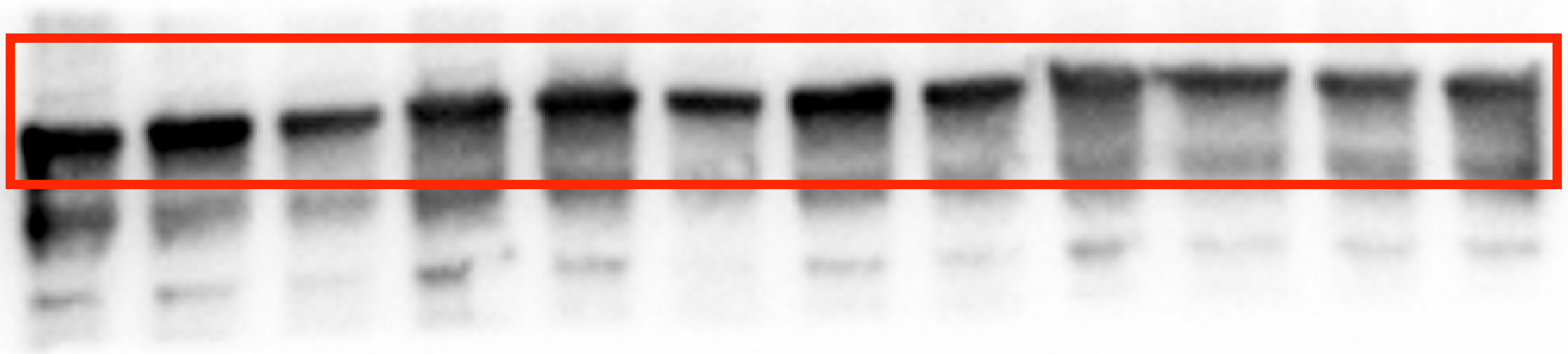

Supplement: Supplementary file 2 — Supplementary Information 2. [file 41598_2024_60239_MOESM2_ESM.pdf]
