# Supplementary material for: Rho kinase inhibitor Y-27632 downregulates IL-1β expression in mice with experimental autoimmune myocarditis
Source: Sci Rep. 2024 Apr 29;14:9763. doi: 10.1038/s41598-024-60239-8 (PMC11058197; doi:10.1038/s41598-024-60239-8)
Supplement: Supplementary file 3 — Supplementary Information 3. [file 41598_2024_60239_MOESM3_ESM.docx]

**Supplemental materials**

Table S1 BW, HW and HW/e-BW of mice in the control and EAM groups

| Indexes | Control group  (Mean ± SD) | EAM group  (Mean ± SD) |
| --- | --- | --- |
| s-BW(g) | 22.62 ± 2.06 | 22.94 ± 1.53 |
| e-BW(g) | 28.77± 3.24 | 24.08 ± 0.84 |
| c-BW(g) | 6.15 ± 2.17 | 1.14 ± 1.48 |
| HW(g) | 0.16 ± 0.01 | 0.21 ± 0.03 |
| HW/e-BW(g/g) | 0.006 ± 0.001 | 0.009 ± 0.001 |

**Note:** body weight, BW; starting body weight, s-BW; eventual body weight, e-BW; change of body weight, c-BW; heart weight, HW; experimental autoimmune myocarditis, EAM.

Table S2 BW, HW and HW/e-BW of mice in the saline and Y-27632 2HCl groups

| Indexes | Saline group  (Mean ± SD) | Y-27632 2HCl group  (Mean ± SD) |
| --- | --- | --- |
| s-BW(g) | 22.86 ± 1.07 | 22.65 ± 1.52 |
| e-BW(g) | 23.92 ± 1.57 | 28.07 ± 2.08 |
| c-BW(g) | 1.06 ± 1.03 | 5.42 ± 1.51 |
| HW(g) | 0.21 ± 0.03 | 0.17 ± 0.03 |
| HW/ e-BW(g/g) | 0.009 ± 0.001 | 0.006 ± 0.001 |

**Note:** body weight, BW; starting body weight, s-BW; eventual body weight, e-BW; change of body weight, c-BW; heart weight, HW; experimental autoimmune myocarditis, EAM.

Table S3 The primer sequences for qRT-PCR

| Primers | Primer sequences (5' 3') |
| --- | --- |
| Il-1β F | GTGTCTTTCCCGTGGACCTTC |
| Il-1β R | TCATCTCGGAGCCTGTAGTGC |
| Tl2 F | TCAGTCCCAAAGTCTAAAGTCG |
| Tl2 R | ATCTACGGGCAGTGGTGAAAA |
| Tl4 F | TTATTCAGAGCCGTTGGTGTATC |
| Tl4 R | CTCCCATTCCAGGTAGGTGTT |
| Notch1 F | CGCAAGCACCCAATCAAG |
| Notch1 R | TAGGAAGGCAGCCACATCG |
| Hes1 F | GAGAAGAGGCGAAGGGCAAG |
| Hes1 R | ACGCTCGGGTCTGTGCTGA |
| Jag2 F | CAATGACTTCTACTGTGCCTGTGAC |
| Jag2 R | CTGCTGTTCTTGGCGATGGT |
| Dil1 F | AATGGAGGACGATGTTCAGATAA |
| Dil1 R | ACAGGTAAGAGTTGCCGAGGT |
| Gapdh F | GTGTCTTTCCCGTGGACCTTC |
| Gapdh R | TCATCTCGGAGCCTGTAGTGC |
